# X-linked adrenoleukodystrophy presenting as progressive ataxia and pure cerebellar involvement

**DOI:** 10.1590/0004-282X-ANP-2020-0294

**Published:** 2021-05-01

**Authors:** Marianna Pinheiro Moraes de Moraes, Augusto Bragança Reis Rosa, Cristina Saade Jaques, Victor Hugo Rocha Marussi, José Luiz Pedroso, Orlando Graziani Barsottini

**Affiliations:** 1 Universidade Federal de São Paulo Unidade de Ataxia Departamento de Neurologia São Paulo SP Brazil Universidade Federal de São Paulo, Unidade de Ataxia, Departamento de Neurologia, São Paulo SP, Brazil.; 2 Hospital Beneficência Portuguesa de São Paulo Departamento de Neurorradiologia São Paulo SP Brazil Hospital Beneficência Portuguesa de São Paulo, Departamento de Neurorradiologia, São Paulo SP, Brazil.; 3 Universidade Federal de São Paulo Divisão de Neurorradiologia Departamento de Radiologia São Paulo SP Brazil Universidade Federal de São Paulo, Departamento de Radiologia, Divisão de Neurorradiologia, São Paulo SP, Brazil.

**Figure 1:**
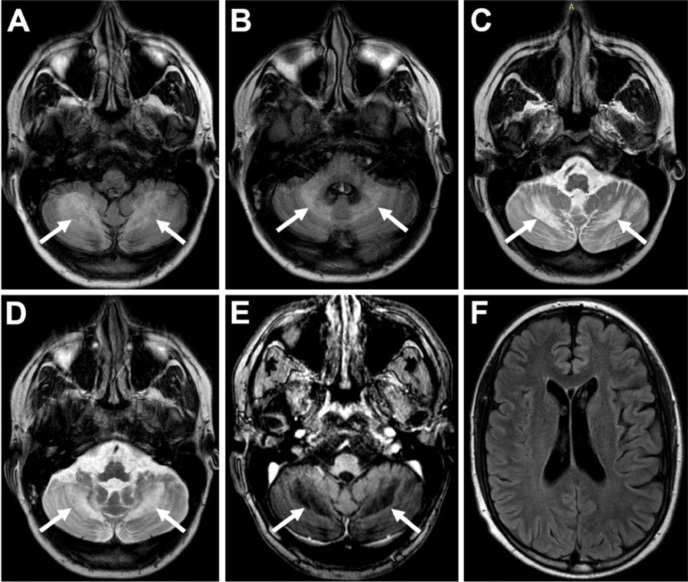
Patient with pure cerebellar ataxia related to X-linked adrenoleukodystrophy. Axial FLAIR-weighted brain MRI shows symmetrical cerebellar white matter and middle cerebellar peduncles hyperintense signal, besides pontocerebellar atrophy (A and B). Axial T2-weighted brain MRI discloses bilateral cerebellar white matter changes (C and D). Axial T1-weighted brain MRI reveals hypointense signal in cerebellar white matter (E). Axial FLAIR-weighted brain MRI shows no supratentorial abnormalities (F).

A 27-year-old man presented with a two-year history of progressive ataxia. Family history was unremarkable. Examination revealed ataxia and alopecia. Serum cortisol levels were low, suggesting adrenal insufficiency. Brain magnetic resonance imaging (MRI) disclosed cerebellar white matter involvement (Figure 1). Exome sequencing showed homozygous mutations (c.268del p.Glu90Argfs*13) in the *ABCD1* gene and confirmed X-linked adrenoleukodystrophy (X-ALD).

Several forms of hereditary ataxias remain undetermined, despite being largely investigated. Whole-exome sequencing is a useful diagnostic approach for undetermined ataxias^1^. Adult-onset X-ALD usually presents with behavioral changes, pyramidal signs, and white matter changes. Pure cerebellar white matter changes with progressive cerebellar ataxia are uncommon in X-ALD^2^.
